# AMPK integrates metabolite and kinase-based immunometabolic control in macrophages

**DOI:** 10.1016/j.molmet.2022.101661

**Published:** 2022-12-28

**Authors:** Iain R. Phair, Raid B. Nisr, Andrew J.M. Howden, Magdalena Sovakova, Noor Alqurashi, Marc Foretz, Douglas Lamont, Benoit Viollet, Graham Rena

**Affiliations:** 1 Cellular and Systems Medicine, School of Medicine, University of Dundee, Dundee, DD1 9SY, UK; 2 Cell Signalling and Immunology, School of Life Sciences, University of Dundee, Dundee, DD1 5EH, UK; 3 Université Paris Cité, Institut Cochin, CNRS, INSERM, F-75014 Paris, France; 4 Centre for Advanced Scientific Technologies, School of Life Sciences, University of Dundee, Dundee, DD1 5EH, UK

**Keywords:** AMPK, Metformin, A769662, Macrophages, Immunometabolism, Itaconate, Prostaglandins, Glucose, Cholesterol, Arginine

## Abstract

**Objective:**

Previous mechanistic studies on immunometabolism have focused on metabolite-based paradigms of regulation, such as itaconate. Here, we, demonstrate integration of metabolite and kinase-based immunometabolic control by AMP kinase.

**Methods:**

We combined whole cell quantitative proteomics with gene knockout of AMPKα1.

**Results:**

Comparing macrophages with AMPKα1 catalytic subunit deletion with wild-type, inflammatory markers are largely unchanged in unstimulated cells, but with an LPS stimulus, AMPKα1 knockout leads to a striking M1 hyperpolarisation. Deletion of AMPKα1 also resulted in increased expression of rate-limiting enzymes involved in itaconate synthesis, metabolism of glucose, arginine, prostaglandins and cholesterol. Consistent with this, we observed functional changes in prostaglandin synthesis and arginine metabolism. Selective AMPKα1 activation also unlocks additional regulation of IL-6 and IL-12 in M1 macrophages.

**Conclusions:**

Together, our results validate AMPK as a pivotal immunometabolic regulator in macrophages.

## Highlights

• Immunometabolism is increasingly seen as an important regulator of inflammation.
• AMPK gene knockout leads to a striking hyperpolarisation of M1 macrophages.
• AMPK controls M1 polarisation by integrating control of several metabolic pathways.
• Our results validate AMPK as a pivotal immunometabolic regulator.

## 1 Introduction

In ‘classic’ polarisation, macrophages undergo pro-inflammatory M1 activation in response to cues including Toll Like Receptor (TLR) agonists and IFNγ but in contrast, in response to agents including IL-4, IL-10 or IL-13, they become M2 macrophages, which are generally thought of as having anti-inflammatory or tissue repair actions [1]. Aberrant regulation of macrophage polarisation and activation is suspected to underlie pathology in metabolic illness such as diabetes and cardiovascular disease [2,3] but the regulatory paradigms underlying immunometabolic control are still being determined. During an early evolutionary phase, regulation of immunometabolic enzymes is likely to have occurred in response to nutrients and metabolites [4] such as itaconate, which has been shown to mediate suppression of M1 cytokines, IL-1β and TNF-α, as well as suppression of the M1 marker iNOS [5]. Later on, enzymes such as AMP-activated protein kinase (AMPK) evolved to provide additional layers of regulation/signal integration, based on enzyme-mediated post-translational modification of target proteins [4]. AMPK is recognised as a critical regulator of cellular metabolism through sensing of AMP levels leading to activation of catalytic processes to generate ATP [6,7], with orthologues from yeast to mammals; however data on its contribution to immunometabolic control is not systems-wide and has sometimes depended on non-specific pharmacological agents, particularly AICAR.

In mammals, AMPK is a heterotrimeric protein complex composed of a catalytic α subunit, an AMP-binding regulatory γ subunit, and a scaffolding β subunit. Binding of AMP to the γ subunit induces allosteric changes in AMPK, resulting in the exposure of Thr172 in the catalytic loop of the α subunit. Phosphorylation of this residue increases kinase activity 100-fold [7]. In macrophages, α1 is the most abundant catalytic subunit [8] and AMPK is essential for controlling rates of fatty acid oxidation as well as the reduction of inflammation [9]. Functionally, AMPK is thought to act as a negative regulator of LPS-stimulated inflammatory M1 macrophage polarisation [[10], [11], [12], [13]]. Myeloid-specific deletion of AMPKα1 or AMPKα2 exacerbated atherogenesis in *LDLR*^*−/−*^ and *APOE*^*−/−*^ mouse models of atherosclerosis respectively [14,15]; however, was without effect in a PCSK-9 western diet model of atherosclerosis [16]. In addition, AMPKα1 has been shown to play a crucial role in skewing macrophages towards a pro-resolution M2 phenotype during muscle regeneration [12]. AMPK activation was also shown to prevent the development of long-term atherosclerosis in ApoE-deficient mice by reducing the number of atheromata macrophages [17], as well as enhancing the anti-atherogenic effects of HDLs [18]. The AMPK activator metformin is understood to suppress cardiovascular inflammation in humans and regress left ventricular hypertrophy irrespective of diabetes status, supporting possible immunometabolic therapeutic properties of this drug [19,20]. More broadly anti-inflammatory drugs have been shown to have efficacy in cardiovascular disease [21], suggesting that immunometabolic regulation may be an important new modality for cardiovascular disease therapy. Here, we have used quantitative mass spectrometry to systematically define the role of AMPKα1 in macrophage function. The current study linked quantitative mass spectrometry, AMPK gene knockout and a selective activator of AMPKα1-containing complexes, as a systematic platform to define the roles of basal AMPKα1 activity and activation in immunometabolic control of macrophage function.

## 2 Materials and methods

### 2.1 Mice

Wild-type C57BL/6J mice were obtained from Charles River. For experiments using AMPKα1 KO mice, these animals and their littermate controls have been described previously [22]. Briefly, *PRKAA1*^*−/−*^ and *PRKAA1*^*+/+*^ mice produced in the F1 generation of breedings of heterozygous parents were crossed with *PRKAA1*^*+/−*^ and *PRKAA1*^*+/+*^ respectively to generate homozygous and control mice. Animals were maintained under a 12 h:12 h light:dark cycle (holding room lights on at 06:00 and off at 18:00) at 22 ± 1 °C and 50% humidity. Mice had ad libitum access to standard chow diet (7.5% fat, 75% carbohydrate, and 17.5% protein by energy [RM1 diet; Special Diet Services]) and water. All animal care protocols and procedures were performed in accordance with current regulations. Colonies were maintained under specific pathogen-free conditions, work was approved by local ethical review and carried out subject to a home office licence.

### 2.2 BMDM culture

Primary bone marrow-derived macrophages (BMDMs) were generated as previously described. Briefly, bone marrow was flushed from the femurs and tibia of 6- to 12-week-old C57BL/6J mice and the bone marrow was passed through a 100 μm cell strainer (Greiner). Macrophages were differentiated for 7 days on bacterial grade plastic in BMDM media: DMEM (Gibco) supplemented with 20% (v/v) L929-conditioned media as a source of M-CSF, 2 mM Glutamine (Gibco), 10% (v/v) foetal calf serum (Labtech), 50 μM 2-mercaptoethanol, 10 mM HEPES (Sigma), 100 U/ml Penicillin (Gibco) and 100 μg/ml Streptomycin (Gibco). On day 7, cells were harvested and replated at a density of 500,000 cells/ml on tissue culture-treated plastic in fresh BMDM media. BMDMs were stimulated as indicated in legends with 100 ng/ml LPS from *E. Coli*, Serotype O55:B5 (Enzo Life Sciences). Metformin came from Sigma, and C13 was a gift from Prof Grahame Hardie, University of Dundee.

### 2.3 Proteomics sample preparation

Peptides were generated using the S-Trap mini column method (Protifi) [87]. Briefly, cells were lysed in 4% SDS, 50 mM TEAB pH 8.5, 10 mM TCEP at room temperature. Samples were transferred to Protein LoBind tubes (Eppendorf), boiled using a ThermoMixer (5 min at 95 °C with 500 rpm on shaker), followed by sonication with a BioRuptor (15 cycles of 30 s on/30 s off). Protein concentration was determined using the EZQ Protein Quantitation Kit (Invitrogen) as per manufacturer’s instructions. Samples were alkylated with 20 mM Iodoacetamide (IAA) in the dark at room temperature for 1 h. Lysates were then acidified by the addition of 1.2% phosphoric acid, then mixed by vortexing. S-Trap binding buffer (90% HPLC-grade methanol containing 100 mM TEAB pH 7.1) was added to lysates at a 7:1 ratio. Samples were then loaded on S-Trap mini columns, then centrifuged at 4000 *g* for 30 s. Columns were then washed five times with S-Trap binding buffer. Digestion buffer (50 mM ammonium bicarbonate containing trypsin (Promega) at a 1:20 ratio (trypsin:protein)) was added to the top of the column and incubated at 47 °C without shaking for 2 h. Peptides were then eluted by addition and sequential centrifugation at 4000*g* of 80 μl of digestion buffer, 80 μl of 0.2% formic acid, and 80 μl of 50% acetonitrile containing 0.2% formic acid. Eluted peptides were dried down using a SpeedVac (GeneVac), then reconstituted in 1% formic acid by incubating on a ThermoMixer at 30 °C for 1 h with shaking at 1000 rpm.

### 2.4 LC-MS analysis

Peptides were analysed on a Q Exactive™ plus, Mass Spectrometer (Thermo Scientific) coupled to a Dionex Ultimate 3000 RS nano (Thermo Scientific). The following LC buffers were used: buffer A (0.1% formic acid in Milli-Q water (v/v)) and buffer B (80% acetonitrile and 0.1% formic acid in Milli-Q water (v/v)). An equivalent of 1.5 μg of each sample was loaded at 10 μL/min onto a μPAC trapping C18 column (Pharmafluidics). The trapping column was washed for 6 min at the same flow rate with 0.1% TFA and then switched in-line with a Pharma Fluidics, 200 cm, μPAC nanoLC C18 column. The column was equilibrated at a flow rate of 300 nl/min for 30 min. The peptides were eluted from the column at a constant flow rate of 300 nl/min with a linear gradient from 1% buffer B to 3.8% buffer B in 6 min, from 3.8% B to 12.5% buffer B in 22 min, from 12.5% buffer B to 41.3% buffer B within 95 min and then from 41.3% buffer B to 61.3% buffer B in 23 min. The gradient was finally increased from 61.3% buffer B to 100% buffer B in 10 min, and the column was then washed at 100% buffer B for 10 min. Two blanks were run between each sample to reduce carry-over. The column was kept at a constant temperature of 50 °C. Q-exactive plus was operated in positive ionization mode using an easy spray source. The source voltage was set to 2.2 Kv and the capillary temperature was 275 °C. Data were acquired in Data Independent Acquisition Mode as previously described [23], with some modifications. A scan cycle comprised of a full MS scan (*m*/*z* range from 345 to 1155), resolution was set to 70,000, AGC target 3 × 10^6^, maximum injection time 200 ms. MS survey scans were followed by DIA scans of dynamic window widths with an overlap of 0.5 Th. DIA spectra were recorded at a resolution of 17,500 at 200 *m*/*z* using an automatic gain control target of 3 × 10^6^, a maximum injection time of 55 ms and a first fixed mass of 200 *m*/*z*. Normalised collision energy was set to 25% with a default charge state set at 3. Data for both MS scan and MS/MS DIA scan events were acquired in profile mode.

### 2.5 Mass spectrometry data analysis

Raw mass spectrometry data was processed using Spectronaut (Biognosys) version 15.0.210615.50606 with the DirectDIA option selected. The following parameters were chosen: cleavage rules were set to Trypsin/P, maximum peptide length 52 amino acids, minimum peptide length 7 amino acids, maximum missed cleavages 2. Carbamidomethylation of cysteine was set as a fixed modification while the following variable modifications were selected: oxidation of methionine, deamidation of asparagine and glutamine and acetylation of the protein N-terminus. The FDR threshold for both precursor and protein was set at 1%. Profiling and imputation were disabled. Quant 2.0 was selected. DirectDIA data were searched against a mouse database from Uniprot release 2020 06. This database consisted of all manually annotated mouse SwissProt entries along with mouse TrEMBL entries with protein level evidence and a manually annotated homologue within the human SwissProt database. Estimates of protein copy number per cell were calculated using the histone ruler method [24].

### 2.6 Flow cytometry of macrophages

Following treatments, BMDMs were harvested from cell culture plates using 5 mmol/l EDTA in PBS for 10 min at 37 °C. Cells were washed in FACS buffer (PBS containing 1% (w/v) bovine serum albumin (BSA; Sigma-Aldrich)). Cells were incubated with 10 μg/ml anti-mouse CD16/32 (clone 93, BioLegend) to block Fc receptors, then stained with anti-F4/80-BV421 (1:400; clone BM8, BioLegend) and anti-CD11b-PE (1:400; clone M1/70, BioLegend) in FACS buffer for 20 min at 4 °C. Stained cells were washed and resuspended in FACS buffer and acquired on a BD LSRFortessa (BD Biosciences) using FACSDiva software. Analysis was carried out using FlowJo software. For analysis, live cells were identified by gating based on forward and side scatter.

### 2.7 Immunoblotting

Cells were lysed in 50 mM Tris–HCl (pH 7.5), 1 mM EGTA, 1 mM EDTA, 1 mM sodium orthovanadate, 50 mM sodium fluoride, 1 mM sodium pyrophosphate, 0.27 M sucrose, 1% (v/v) Triton X-100, 0.1% (v/v) 2-mercaptoethanol, and cOmplete EDTA-free Protease Inhibitor Cocktail tablets (Roche). Lysates were clarified by centrifugation (20800 *g* for 10 min at 4 °C) and supernatants snap-frozen and stored at −80 °C. Protein concentration was determined with Coomassie Protein Assay Reagent (Thermo Scientific). Proteins were separated on 4–12% gradient Bis-Tris polyacrylamide gels (Invitrogen), and immunoblotting carried out using standard techniques. Antibodies recognising total acetyl-CoA carboxylase (Cat #3676), total AMPKα (#5832), phospho-acetyl-CoA carboxylase S79 (#11818), phospho-AMPKα T172 (#2535), and GAPDH (#5174) were from Cell Signaling Technology and used at a dilution of 1 in 1000. Anti-rabbit horseradish peroxidase (#7074) was from Cell Signaling Technology.

### 2.8 BMDM mitochondrial bioenergetics and cellular acidification rate

BMDMs (5 × 10^4^ cells/well) were seeded in Seahorse XF24 culture plates followed by stimulation with 100 ng/ml LPS for 24 h. Cells were washed 3 times with Seahorse XF base medium (Agilent) containing 5 mM glucose and 10 mM HEPES, with pH adjusted to 7.4. Cells were then incubated in this media at 37 °C without CO_2_ for 1 h. The assay involved 5 measurement cycles of basal oxygen consumption rate (OCR) and extracellular acidification rate (ECAR) followed by sequential injections of 1 μM oligomycin to determine the ATP linked respiration (oligomycin sensitivity) and proton leak (oligomycin resistance), 1 μM FCCP to determine the maximal respiration and then a mix of 1 μM Rotenone and 2 μM antimycin A to measure the non-mitochondrial respiration. After each of these injections, four measurement cycles of OCR and ECAR were taken.

### 2.9 Measurement of lactate production

BMDMs were stimulated with 100 ng/ml LPS for times stated and cell culture supernatants were harvested. Lactate concentration was determined using the Lactate-Glo Assay (Promega) according to the manufacturer’s protocols.

### 2.10 Analysis of nitric oxide production

BMDMs were stimulated with 100 ng/ml LPS for times stated and cell culture supernatants were harvested. Nitric oxide (NO) production was measured using a colorimetric Nitric Oxide Assay Kit (Abcam) according to the manufacturer’s protocols.

### 2.11 Analysis of PGE_2_ production

BMDMs were stimulated with 100 ng/ml LPS for times stated and cell culture supernatants were harvested. Prostaglandin E_2_ (PGE_2_) production was measured using a PGE_2_ enzyme-linked immunosorbent assay (ELISA) kit from Enzo Life Sciences according to the manufacturer’s protocols.

### 2.12 Analysis of cytokine production

BMDMs were stimulated with 100 ng/ml LPS for times stated and cell culture supernatants were harvested. Levels of IL-6, IL-10 and IL-12p40 present in the media were determined via a multiplex Luminex-based method (Bioplex, Bio-Rad) using the Bio-Plex 200 system (Bio-Rad).

### 2.13 Statistical analyses

For statistical analysis of proteomics data, four biological replicates were generated, and *P* values were calculated using a two-tailed *t*-test assuming unequal variance on log normalised “copy numbers per cell” values. For expression of individual proteins, data are presented as the mean ± s.d. Differences were considered statistically significant if *p* < 0.05. Heat maps were generated using the Morpheus tool from the Broad Institute (http://software.broadinstitute.org/morpheus). For Gene Ontology (GO) Term enrichment analysis, GO terms enriched in proteins with statistically significant changes in expression were identified using the functional annotation tools within DAVID Bioinformatics Resources 6.8, NIAID/NIH (https://david.ncifcrf.gov/). All other data are presented as mean values ± s.d. unless otherwise stated. A Student’s *t*-test (two-tailed, unpaired) was performed in Excel and ANOVA testing in GraphPad Prism.

## 3 Results

As AMPKα1 is the dominant catalytic subunit in both human and murine macrophages [10], AMPKα1 knockout (KO) bone marrow-derived macrophages (BMDMs) were used to investigate the role of AMPK in macrophage function and activation. Total AMPKα protein expression was lost in AMPKα1 KO BMDMs, and importantly there was no compensatory upregulation of AMPKα2 (Supplementary Fig. 1a). Activation of AMPK either by treatment with metformin or the highly specific α1-isoform activator of AMPK compound 13 (C13), as determined by phosphorylation of the AMPK substrate ACC, was lost in AMPKα1 KO BMDMs (Supplementary Figs. 1a and b). In addition, wild-type and AMPKα1 KO BMDMs expressed comparable levels of macrophage markers F4/80 and CD11b, indicating that macrophage differentiation is not prevented by AMPKα1 deletion (Supplementary Fig 1c-e).

We carried out quantitative proteomic analysis of AMPKα1 KO macrophages and those taken from wild-type littermates. In these experiments we identified 6455 proteins in wild-type and AMPKα1 KO macrophages, and absolute copy numbers were estimated using the proteomic ruler method which uses the mass spectrometry signal of histone proteins as an internal standard (Wisniewski et al., 2014). Notably, expression of both AMPKβ1 and AMPKγ1 were decreased in AMPKα1 KO macrophages, suggestive that AMPKα1 is important for stabilisation of the scaffolding and regulatory subunits (Supplementary Fig 1f). We first analysed the impact of AMPKα1 deletion on the proteome of naïve BMDMs. Using a fold change cut off >2 and *P* value < 0.05 we observed increases in the expression of 160 proteins in AMPKα1 KO BMDMs, whilst 48 proteins were downregulated, suggestive that basal AMPK activity may play a role in shaping macrophage differentiation (Supplementary Fig. 2a). Notably, AMPKα1 deletion reduced the expression of markers of anti-inflammatory M2 macrophages Ym1, Mannose receptor C-type 1 (MRC1), CD36, CD301 and Glutamine synthetase (GS) (Supplementary Fig. 2b and c), as well as CD169, a marker for M2-like tumour-associated macrophages [25]. In contrast, deletion of AMPKα1 did not affect the expression of proteins associated with pro-inflammatory M1 macrophages. We also found that expression of ApoE, which promotes cholesterol efflux, was almost completely abolished in that genotype (Supplementary Fig. 2d). ApoE is a reported target gene of LXRs, and whilst we did not detect expression of LXRs in our dataset, deletion of AMPKa1 resulted in decreases in the expression of a range of other known LXR target genes (Supplementary Fig. 2e).

Additionally, the expression of key nutrient transporters was enhanced in AMPKα1 KO macrophages, including those for system A + N (SNAT1/2), system L (4F2, LAT1/2), cationic amino acids (CAT1/2), glucose (GLUT1), lactate (MCT4) and iron (NRAMP1) (Supplementary Fig. 3).

### 3.1 AMPKα1 knockout macrophages exhibit M1 hyperpolarisation

Next, we investigated the impact of AMPKα1 KO on LPS-stimulated activation of macrophages. LPS treatment of both wild-type and AMPKα1 knockout macrophages led to >2-fold increases in the expression of 382 and 318 proteins respectively (Supplementary Fig. 4a and b). In a GO term enrichment analysis of biological processes proteins enhanced by LPS treatment in both WT and AMPKα1 KO macrophages were annotated as associated with processes associated with innate immune responses (Supplementary Fig. 4c).

**Figure 1:**
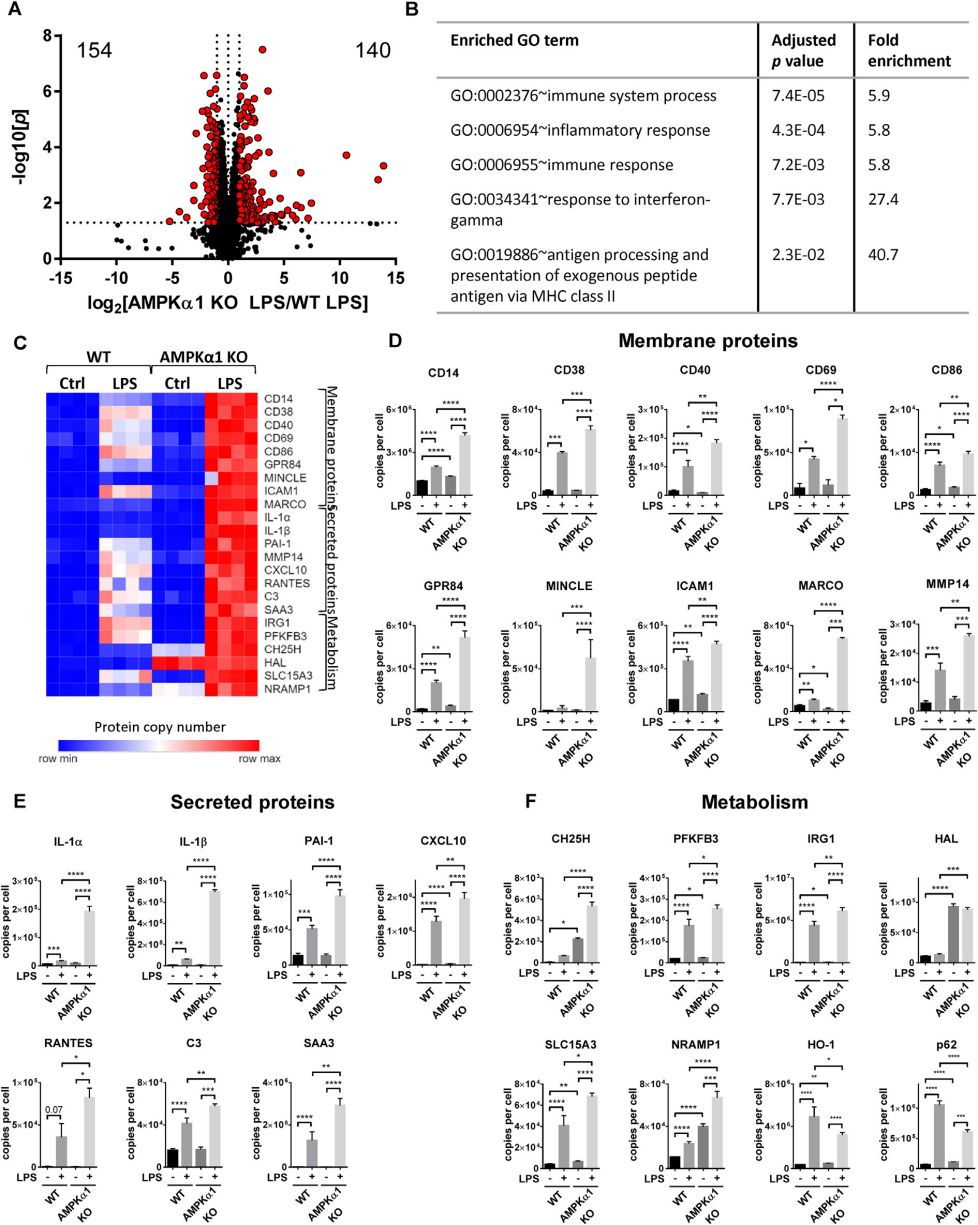
**Deletion of AMPKα1 drives dynamic changes in the proteome of LPS-stimulated macrophages**. (A) Volcano plot shows log2 fold change in mean protein copy numbers per cell between WT and AMPKα1 KO BMDMs stimulated for 24 h with 100 ng/ml LPS. Proteins highlighted in red have a significant (*p* < 0.05) fold change <0.5 or >2. (B) The top five enriched biological process GO terms for proteins expressed >2-fold higher (*p* < 0.05) in LPS-stimulated macrophages lacking AMPKα1. (C) Heat maps showing proteins associated with M1 macrophages in control and LPS-stimulated BMDMs from WT or AMPKα1 KO mice. Relative protein abundance is graded from low (blue) to high (red). (D–F) Mean protein copy number per cell are shown for (D) membrane proteins (CD14, CD38, CD40, CD69, CD86, GPR84, MINCLE, ICAM-1, MARCO and MMP-14), (E) secreted proteins (IL-1α, IL-1β, PAI-1, CXCL10, RANTES, C3 and SAA3) and (F) proteins implicated in metabolism (CH25H, PFKFB3, IRG1, HAL, SLC15A3, NRAMP1, HO-1 and p62). Bar charts represent the mean and standard deviation of results from biological quadruplicates. *P* values were calculated as described in Methods; a *p* < 0.05 is represented by ∗, *p* < 0.01 by ∗∗, *p* < 0.001 by ∗∗∗, and *p* < 0.0001 by ∗∗∗∗. (For interpretation of the references to color in this figure legend, the reader is referred to the Web version of this article.)

In a direct comparison between the proteomes of LPS-stimulated wild-type and AMPKα1 KO macrophages, most proteins did not alter expression levels between genotypes (6161 proteins); however, there were increases in the expression of 140 proteins in LPS-stimulated AMPKα1 KO BMDMs compared to WT, and decreases in the expression of 154 proteins (Figure 1A). Many of the proteins with strongly increased expression in AMPKα1 KO macrophages compared to WT were associated with inflammation (Figure 1B) and the proteins were not regulated differently in the two genotypes in the absence of LPS stimulation. Further investigation of these proteins revealed that a repertoire of M1 surface markers, secreted proteins and metabolic proteins were enhanced in LPS-stimulated AMPKα1 KO BMDMs compared to WT (Figure 1C). Compared with wild-type, there were increases in expression of LPS-stimulated M1 costimulatory markers, including CD38 [26], CD40, and CD86, as well as the early activation marker CD69 (Figure 1D). The expression of receptors associated with inflammatory macrophages was also enhanced by AMPKα1 deletion, including the TLR co-receptor CD14 [27] which was ∼2-fold higher in AMPKα1 KO macrophages compared to wild-type. Similar to previous studies [28], the expression of medium-chain fatty acid receptor GPR84 was strongly enhanced by LPS in wild-type macrophages, however expression was ∼2.5-fold higher in AMPKα1 KO macrophages. The fungal receptor MINCLE [29] and the scavenger receptor MARCO [30,31] which are both hallmarks of M1 macrophages, were also robustly enhanced in LPS-stimulated macrophages lacking AMPKα1 compared to WT. In addition, we observed an increase in Intercellular Adhesion Molecule (ICAM)-1 expression in LPS-stimulated AMPKα1 KO macrophages, which has also been reported as markers of M1 macrophages [32,33]. Stimulation with LPS also enhanced Matrix metalloproteinase (MMP)-14 expression, a membrane bound collagenase that has been shown to play a key role in angiogenesis [34,35], which was exacerbated in AMPKα1-deficient macrophages.

The increase in levels of M1 markers in AMPKα1 KO macrophages was also observed in levels of LPS-stimulated cytokines, chemokines and other secreted proteins associated with acute inflammatory responses (Figure 1E). Previous studies have shown that LPS-stimulated IL-1β expression is robustly increased in macrophages deficient of AMPK activity [36]. Similarly, we observed a ∼10-fold increase in IL-1β expression in AMPKα1 KO macrophages, and we also observed a similar increase in IL-1α expression. Chemokines produced by inflammatory macrophages were also enhanced in AMPKα1 KO cells (CXCL10 and RANTES). There is strong evidence that elevated PAI-1 levels are associated with both CVD and metabolic syndrome [[37], [38], [39], [40], [41]]. Treatment with LPS promoted the expression of PAI-1, which was higher in macrophages deficient in AMPKα1 than WT. We also observed elevated levels of complement component 3 (C3) in AMPKα1 KO cells, which plays a critical role in the activation of both the classical and alternative complement systems [42,43]. Acute phase serum amyloid A (SAA) proteins are highly induced during acute phase inflammatory responses, driving production of inflammatory cytokines and metalloproteases in macrophages and smooth muscle cells. *SAA3* is thought to be a pseudogene in humans, though SAA3 is expressed as a functional protein in mice. SAA3 was robustly induced by LPS treatment, and was observed to be ∼2-fold higher in AMPKα1 KO macrophages.

Changes in the expression of proteins associated with metabolic functions were also observed in AMPKα1 KO BMDMs (Figure 1F). Stimulation of macrophages with LPS is associated with the upregulation of glycolysis, driven by an increase in the expression of the glycolytic activator 6-phosphofructo-2-kinase/fructose-2,6-biphosphatase 3 (PFKFB3) [44]. Immune-Responsive Gene 1 (IRG1) is also robustly upregulated in M1 macrophages [45], leading to an increase in the production of the endogenous anti-inflammatory metabolite itaconate [5]. Both IRG1 and PFKFB3 were stimulated by LPS treatment, which was enhanced in AMPKα1 KO macrophages. Cholesterol 25-hydroxylase (CH25H), which catalyses 25-hydroxycholesterol (25-HC) production, was also expressed at higher levels in both unstimulated and LPS-stimulated AMPKα1-deficient cells. The expression of proteins associated with histidine metabolism were also impacted by AMPKα1 deletion, with expression of histidine ammonia-lyase (HAL) elevated in AMPKα1 KO BMDMs, irrespective of treatment with LPS. We also observed robust upregulation of the histidine transporter SLC15A3 by LPS stimulation, which was >50% increased in AMPKα1 KO BMDMs compared to WT. Expression of the iron transporter NRAMP1 is also enhanced in M1 macrophages [46]; here we observed elevated expression of NRAMP1 in both unstimulated and LPS-stimulated AMPKα1 KO macrophages. p62 is a protein with many functions, including as a classical receptor of autophagy. Consistent with previous studies, treatment of WT macrophages with LPS upregulated p62, however this was lower in AMPKα1 KO BMDMs. Stimulation with LPS also increases expression of Heme Oxygenase (HO)-1, a protein with cytoprotective and anti-oxidant properties; however, HO-1 was expressed at lower levels in AMPKα1-deficient macrophages.

## 4 Role of AMPKα1 in LPS-stimulated metabolic shift from OXPHOS to glycolysis

**Figure 2:**
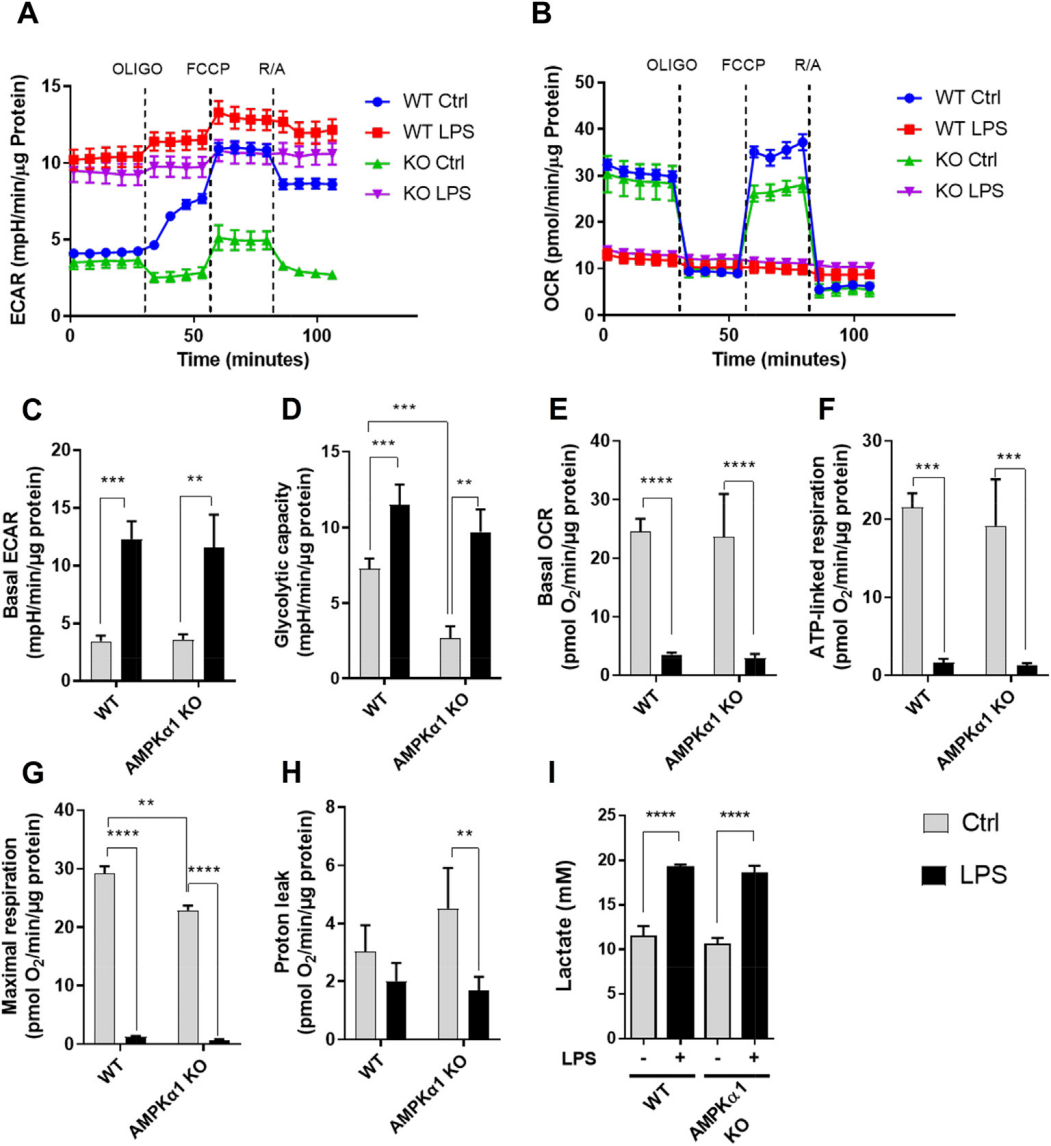
**Metabolic characteristics of AMPKα1-deficient macrophages**. (A–I) Wild-type or AMPKα1 KO BMDMs were stimulated ±100 ng/ml LPS for 24 h. (A–H) Shown are representative Seahorse traces for (A) extracellular acidification rate (ECAR) and (B) oxygen consumption rate (OCR) in which 1 μM oligomycin, 1 μM FCCP and a mix of 1 μM rotenone and 2 μM antimycin were added at times indicated by the dotted line. (C) Basal ECAR, (D) glycolytic capacity, (E) basal OCR, (F) ATP-linked respiration, (G) maximal respiration, and (H) proton leak are shown. For (A–H) data were normalised to protein content, and graphs represent the mean and SEM of results from 5 biological replicates. A *p* < 0.05 is indicated by ∗, *p* < 0.01 by ∗∗, *p* < 0.001 by ∗∗∗ and *p* < 0.0001 by ∗∗∗∗ (two-tailed Student’s *t*-test assuming unequal variance). (I) Lactate production was determined as described in Methods. Graphs represent the mean and standard deviation of results from 4 biological replicates. (For interpretation of the references to color in this figure legend, the reader is referred to the Web version of this article.)

As the expression of PFKFB3 and ACOD1 were enhanced in LPS-stimulated AMPKα1-deficient macrophages compared to WT, we next examined the impact of deletion of AMPKα1 on glycolysis. LPS-activated macrophages are characterised by enhanced glycolysis, coupled with a decrease in oxidative phosphorylation (OXPHOS) [47]. Glycolytic rates, as determined by the extracellular acidification rate (ECAR), were similar in both genotypes; however the glycolytic capacity following oligomycin treatment was significantly lower in AMPKα1 KO macrophages (Figure 2A,C,D). This difference was not seen in LPS-stimulated cells, where glycolytic rate was increased in both genotypes. Basal oxidative phosphorylation and ATP-linked respiration were robustly decreased in BMDMs stimulated with LPS (Figure 2B,E,F); however, there were no observed differences between WT and AMPKα1 KO macrophages. Consistent with this, LPS reduced respiratory chain protein content similarly in both genotypes (Supplementary Fig. 5). The maximal respiratory rate was lower in AMPKα1 KO macrophages (Figure 2G) and there was more proton leak in unstimulated cells (Figure 2H). To confirm that AMPK is not required for the LPS-stimulated metabolic switch to glycolysis, we compared lactate production in WT and AMPKα1 KO BMDMs. LPS stimulation induced an increase in lactate production, with no difference between the responses of WT and AMPKα1 KO macrophages (Figure 2I). Together, these data suggest that AMPK is dispensible for the metabolic switch to glycolysis stimulated by LPS.

### 4.1 Effect of AMPKα1 deletion on arginine metabolism

**Figure 3:**
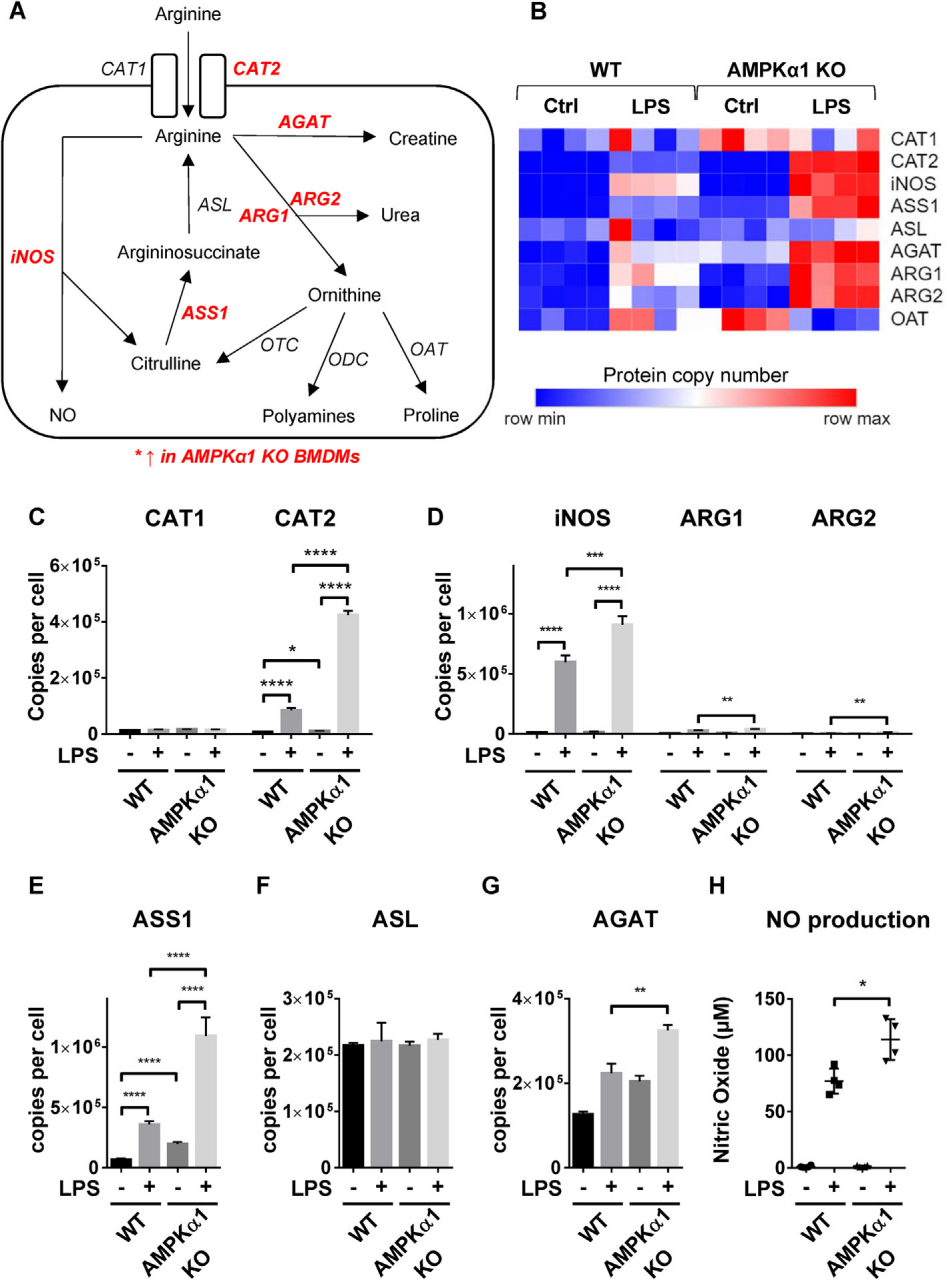
**Arginine metabolic enzymes are enhanced in AMPKα1 KO macrophages**. (A) Schematic of arginine metabolism in macrophages. Proteins with enhanced expression in AMPKα1 KO BMDMs are shown in red italics. (B) Heat maps of proteins associated with arginine metabolism in control and LPS-stimulated BMDMs from WT or AMPKα1 KO mice. Relative protein abundance is graded from low (blue) to high (red). (C–G) Mean protein copy number per cell are shown for (C) CAT1 and CAT2, (d) iNOS, ARG1 and ARG2, (e) ASS1, (f) ASL, and (G) AGAT. Bar charts represent the mean and standard deviation of results from biological quadruplicates. *P* values were calculated as described in Methods; a *p* < 0.05 is represented by ∗, *p* < 0.01 by ∗∗, *p* < 0.001 by ∗∗∗, and *p* < 0.0001 by ∗∗∗∗. (H) WT or AMPKα1 KO BMDMs were stimulated ±100 ng/ml LPS for 24 h, and the levels of nitric oxide (NO) secreted into the media were determined as described in Methods. Graphs represent the mean and standard deviation of results from 4 biological replicates. A *p* < 0.05 is indicated by ∗ (two-tailed Student’s *t*-test assuming unequal variance). (For interpretation of the references to color in this figure legend, the reader is referred to the Web version of this article.)

We next investigated the effect of AMPKα1 deletion on arginine metabolism (Figure 3A), a process that is integrated in the immune response in macrophages. Proteins associated with arginine metabolism were observed to be enhanced in LPS-stimulated AMPKα1 KO BMDMs compared to WT (Figure 3B). Uptake of arginine is promoted in M1 macrophages by increased expression of the arginine transporter CAT2 [48]. Consistent with previous studies we observed an increase in CAT2 expression, the absolute expression of which was much higher than the other cationic amino acid transporter, CAT1 (Figure 3C). We observed a ∼4-fold increase in CAT2 expression in LPS-stimulated macrophages deficient in AMPKα1 compared to WT. In addition, LPS drives the upregulation of inducible nitric oxide synthase (iNOS) in M1 macrophages [49]. iNOS is the rate-limiting enzyme in NO production that metabolises arginine to nitric oxide (NO) and citrulline, the NO in turn being metabolised to reactive nitrogen species [50]. Expression of iNOS was observed to be ∼50% higher in AMPKα1 KO BMDMs compared to WT (Figure 3D). In contrast, M2 macrophages express arginases (ARG1/2), which hydrolyse arginine to ornithine and urea, thus limiting arginine availability for NO synthesis [50]. Expression of both ARG1/2 were also upregulated in LPS-stimulated AMPKα1 KO macrophages compared to WT (Figure 3D), however, iNOS expression was ∼20-fold and ∼75-fold higher than ARG1 and ARG2 respectively. M1 macrophages also have elevated levels of argininosuccinate synthase (ASS1), an enzyme responsible for recycling citrulline to generate arginine which can be further utilised by iNOS for NO production [51]. LPS-stimulated levels of ASS1 were ∼3-fold higher in AMPKα1 KO macrophages (Figure 3E). Argininosuccinate lyase (ASL) completes the recycling of citrulline by converting argininosuccinate to arginine, however neither LPS treatment nor AMPKα1 deficiency had an effect on ASL expression (Figure 3F). The third potential pathway for arginine utilisation is in the generation of creatine. Glycine amidinotransferase (AGAT) is rate-limiting in creatine biosynthesis. AGAT expression was observed to be upregulated in AMPKα1 KO macrophages compared to WT following treatment with LPS (Figure 3G). In agreement with enhanced LPS-stimulated iNOS expression in AMPKα1 KO macrophages compared to WT, AMPKα1 KO macrophages also produced higher levels of NO in response to LPS (Figure 3H).

### 4.2 Role of AMPK in prostaglandin synthesis

**Figure 4:**
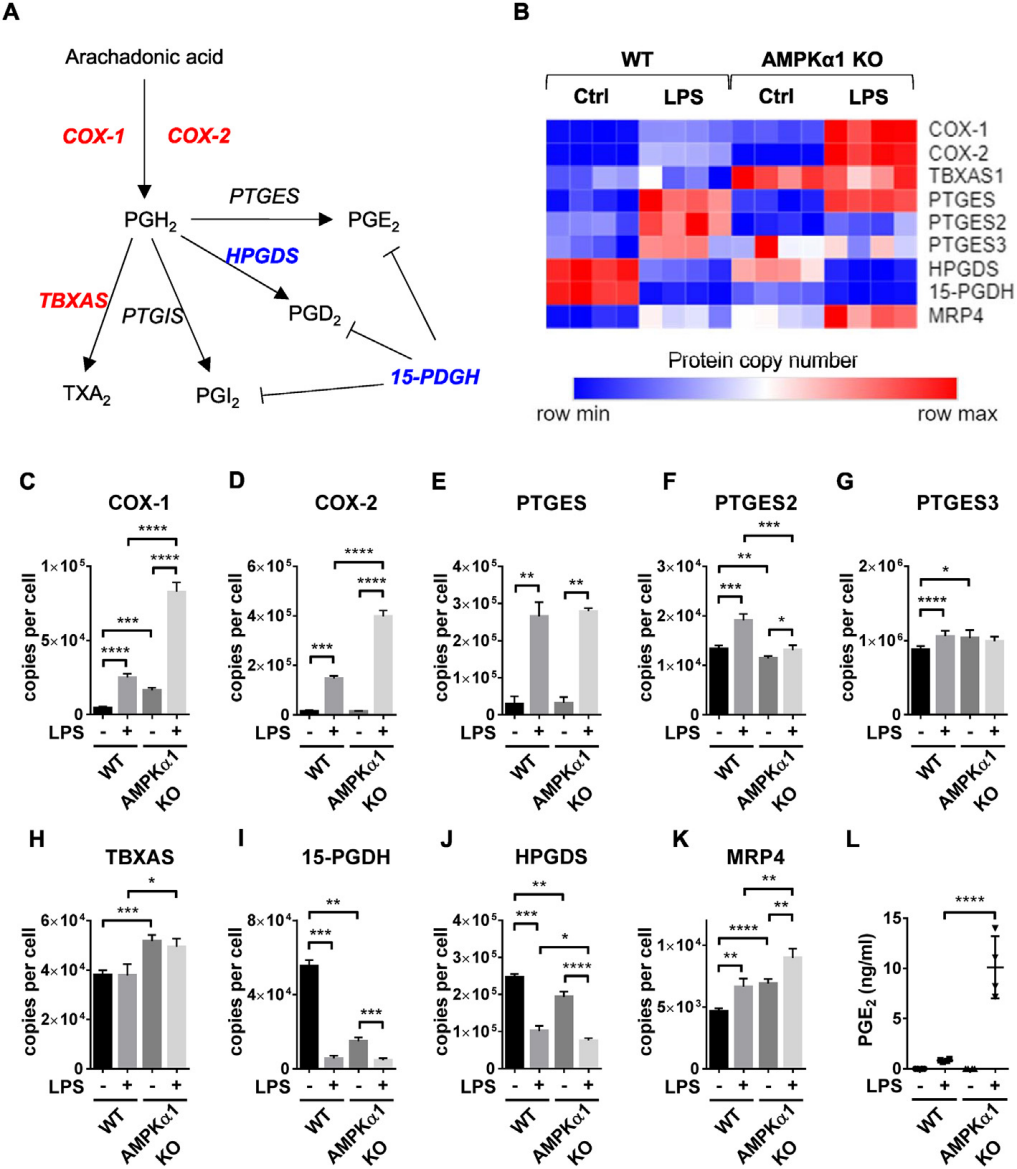
**AMPK regulates prostaglandin synthesis in inflammatory macrophages**. (A) Schematic of prostaglandin synthesis in macrophages. COX-1/2 are rate-limiting enzymes that catalyse the conversion of arachidonic acid to PGH2. PGH2 is subsequently converted to PGE2 by prostaglandin E synthase (PTGES), PGD2 by prostaglandin D synthase (PGDS), PGI2 by prostaglandin I synthase (PTGIS), and TXA2 by thromboxane synthase (TBXAS). Prostaglandin dehydrogenase (15-PGDH) plays a key role in the degradation of prostaglandins. Proteins with increased expression in AMPKα1 KO BMDMs are shown in red, and those with decreased expression are shown in blue. (B) Heat maps of proteins associated with prostaglandin synthesis in control and LPS-stimulated BMDMs from WT or AMPKα1 KO mice. Relative protein abundance is graded from low (blue) to high (red). (C–K) Mean protein copy number per cell are shown for (C) COX-1, (D) COX-2, (E) TBXAS, (F) PTGES, (G) PTGES2, (H) PTGES3, (I) HPGDS, (J) 15-PGDH and (K) MRP4. Bar charts represent the mean and standard deviation of results from biological quadruplicates. *P* values were calculated as described in Methods; a *p* < 0.05 is represented by ∗, *p* < 0.01 by ∗∗, *p* < 0.001 by ∗∗∗, and *p* < 0.0001 by ∗∗∗∗. (L) BMDMs from WT or AMPKα1 KO mice were stimulated with 100 ng/ml LPS for 24 h, and the levels of PGE2 secreted into the media were determined as described in Methods. Graphs represent the mean and standard deviation of results from 4 biological replicates. Two-way ANOVA indicated a significant effect of genotype on PGE2 levels (*F* = 36.32, *p* < 0.0001). (For interpretation of the references to color in this figure legend, the reader is referred to the Web version of this article.)

We next investigated the impact of AMPKα1 deletion on prostaglandin synthesis due to the importance of prostaglandins in the inflammatory response. Prostaglandins are generated from arachidonic acid which is metabolised by the cyclooxygenases COX-1 and COX-2, and subsequently converted to prostaglandin species by prostaglandin synthases (Figure 4A). The expression of proteins involved in prostaglandin biosynthesis were observed to be differentially expressed by treatment with LPS, and differences were observed between WT and AMPKα1 KO cells (Figure 4B). COX-1 and COX-2 are the rate-limiting enzymes in prostaglandin synthesis, and both were >3-fold higher in LPS-stimulated AMPKα1 KO BMDMs compared to WT (Figure 4C,D). There were no observed differences in the expression of LPS-stimulated prostaglandin E synthase (PTGES) or PTGES3 which catalyse the generation of PGE_2_, and whilst there was a significant decrease in PTGES2 expression in AMPKα1 KO macrophages stimulated with LPS compared to WT, PTGES2 was expressed >100-fold and >50-fold lower than PTGES and PTGES3 respectively (Figure 4E,F). Thromboxane A synthase (TBXAS), which converts prostaglandin endoperoxide PGH_2_ to the vasoconstrictor thromboxane A_2_, became constitutively upregulated in AMPKα1 KO cells (Figure 4H). In contrast, Hematopoietic Prostaglandin D Synthase (HPGDS), which metabolises PGH_2_ to PGD_2_, exhibited reduced expression under basal conditions compared to littermates (Figure 4I). 15-PGDH, which is the main prostaglandin and lipoxin-inactivating enzyme [52], was also decreased in the AMPKα1 knockout macrophages [53]. Finally, the prostaglandin efflux transporter MultiDrug Resistance Protein 4 (MRP4) [54] was also upregulated in AMPKα1 KO BMDMs (Figure 4K). In many cell types, Prostaglandin E_2_ (PGE_2_) is the major product of COX-2-mediated metabolism of arachidonic acid, increasing blood flow and vascular permeability during inflammatory responses. In agreement with changes observed in the proteome, LPS-stimulated PGE_2_ production was robustly upregulated in AMPKα1 KO macrophages compared to WT (Figure 4L).

### 4.3 Pharmacological AMPK activation provides an additional control node for cytokine secretion

**Figure 5:**
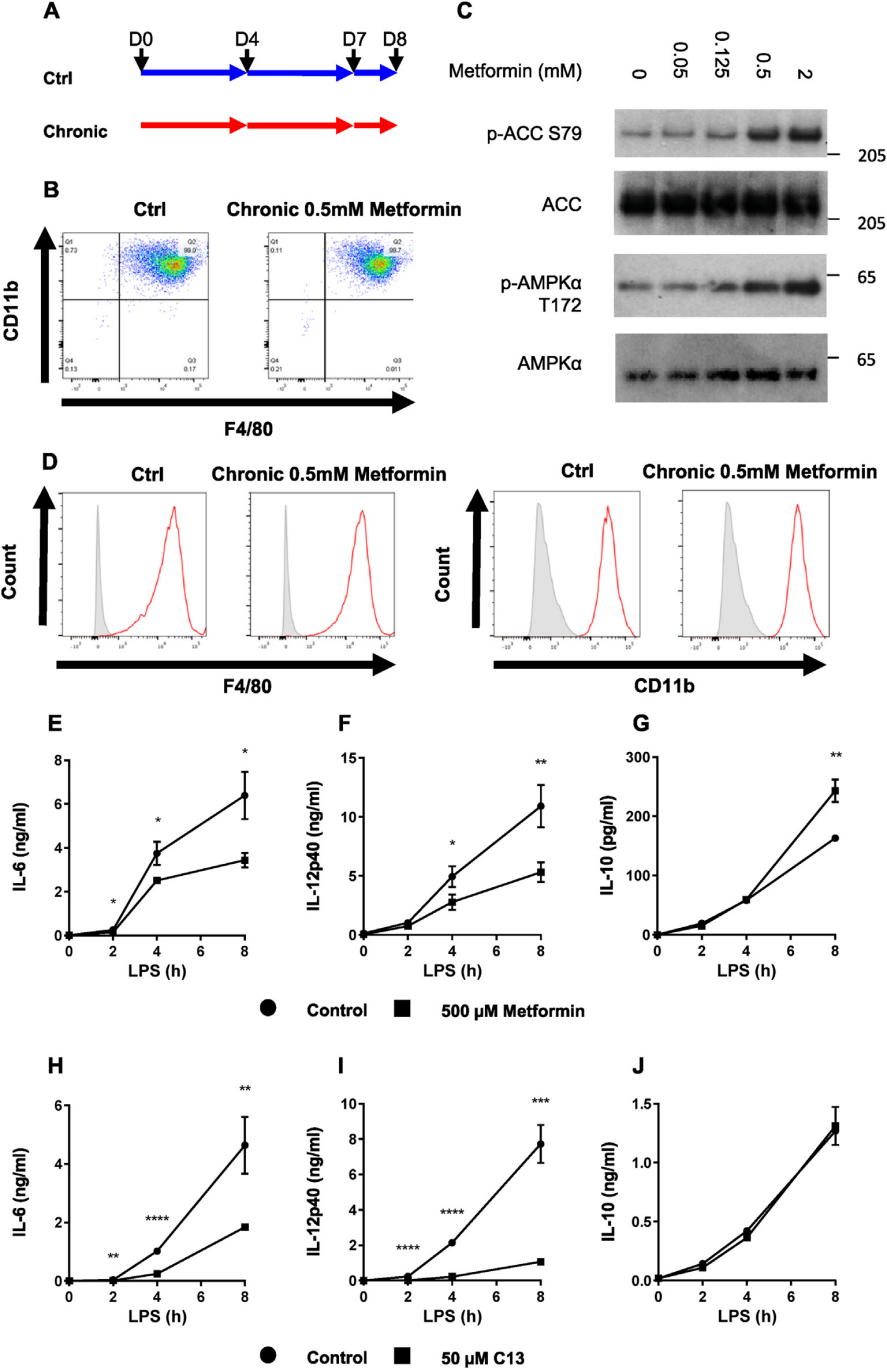
**AMPK agonists reduce pro-inflammatory cytokine production in macrophages**. (A) Schematic of chronic metformin treatment of BMDMs during differentiation. BMDMs were mock treated, or treated with 500 μM metformin on days (D) 0, 4, 7 and 8. (B,D) BMDMs from wild-type mice treated with metformin as described in (A). Cell surface levels of CD11b and F4/80 were determined by flow cytometry. For (D), unstained cells are shown in grey filled histograms, and stained cells in red. (C) BMDMs from wild-type mice were treated ± metformin at doses indicated for 6 h. Cells were then lysed, and levels of phospho-AMPKα T172, AMPKα, phospho-ACC S79 and ACC were determined by immunoblotting. (E–J) BMDMs from wild-type mice were pre-treated with (E–G) 500 μM metformin for 6 h or (H–J) 50 μM C13 for 1 h, then stimulated with 100 ng/ml LPS for times stated. The levels of (E, H) IL-6, (F, I) IL-12p40 and (G, J) IL-10 secreted into the media were determined as described in the Methods. Graphs represent the mean and standard deviation of results from 3 biological replicates. Data are representative of 3 independent experiments. A *p* < 0.05 is indicated by ∗, *p* < 0.01 by ∗∗, *p* < 0.001 by ∗∗∗ and *p* < 0.0001 by ∗∗∗∗ (two-tailed Student’s *t*-test assuming unequal variance). (For interpretation of the references to color in this figure legend, the reader is referred to the Web version of this article.)

Our studies next turned to the role of AMPK activation in cytokine secretion, which we were interested to study due to clinical importance of the AMPK activator metformin. Consistent with the lack of effect of AMPKα1 ablation on CD11b and F4/80 expression (supplementary Fig 1c-e), chronic metformin administration had no effect on these markers either (Figure 5A,B, D).

**Figure 6:**
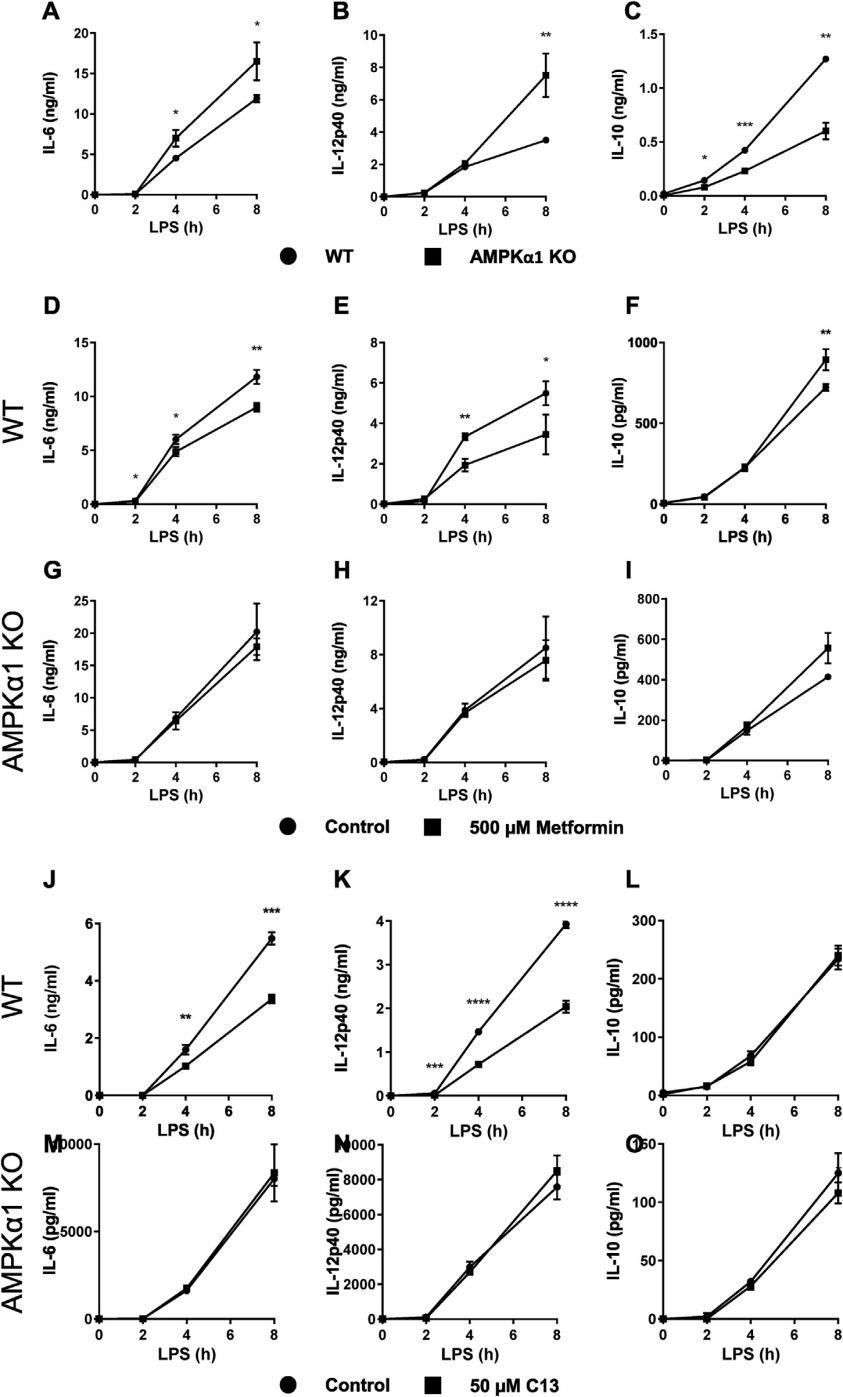
**AMPK activators reduce LPS-stimulated pro-inflammatory cytokine production in macrophages**. (A–C) BMDMs from wild-type or AMPKα1 knockout mice were stimulated with 100 ng/ml LPS for times stated, and the levels of IL-6, IL-12p40 and IL-10 secreted into the media were determined as described in Methods. (D–I) BMDMs from (D–F) wild-type or (G–I) AMPKα1 knockout mice were pre-treated with 500 μM metformin for 6 h, then stimulated with 100 ng/ml LPS for times stated. Levels of IL-6, IL-12p40 and IL-10 secreted into the media were determined as described in Methods. (J–O) BMDMs from (J–L) wild-type or (M–O) AMPKα1 knockout mice were pre-treated with 50 μM C13 for 1 h, then stimulated with 100 ng/ml LPS for times stated. Levels of IL-6, IL-12p40 and IL-10 secreted into the media were determined as described in Methods. Graphs represent the mean and standard deviation of results from 3 biological replicates. Data are representative of 3 independent experiments. A *p* < 0.05 is indicated by ∗, *p* < 0.01 by ∗∗, *p* < 0.001 by ∗∗∗ and *p* < 0.0001 by ∗∗∗∗ (two-tailed Student’s *t*-test assuming unequal variance).

In dose–response experiments we found that metformin consistently induced activation of AMPK at 500 μM (Figure 5C). Previously using higher concentrations, we showed that metformin reduced IL-6 and IL-12 secretion in M1 macrophages [19], whilst others have shown that IL-10 is increased [36]. We observed all three of these responses in our current study (Figure 5E–G). In order to provide further evidence that effects of metformin on IL-6, IL-12 and IL-10 were due to AMPK, we studied effects of compound 13 (C13) [55]. We found that this drug had similar effects to metformin on IL-6 and IL-12 but not on IL-10 (Figure 5H–J). Next, we investigated the effect of AMPKα1 deletion on LPS-stimulated IL-6, IL-12 and IL-10 production to investigate whether AMPK might contribute to regulation of these cytokines. Comparing responses in wild-type and knockout macrophages we found that greater amounts of IL-6 and IL-12 were secreted by knockout macrophages, and less IL-10, consistent with an effect of AMPK on these cytokines, even without pharmacological stimulation of the kinase (Figure 6A–C). When we investigated the action of metformin on macrophages from wild-type littermates, this resulted in less IL-6 and IL-12, and more IL-10 as before (Figure 6D–F). Metformin did not affect IL-6 or IL-12 in AMPKα1 KO macrophages, indicating that these effects of the drug were AMPK-dependent; however, increased secretion of IL-10 was preserved in knockout macrophages (Figure 6G–I). In line with these findings, C13 suppressed IL-6 and IL-12 production (Figure 6J,K), but not IL-10 (Figure 6L). The effects on IL-6 and IL-12 were abrogated in AMPKα1 KO macrophages (Figure 6M,N), whilst similar to the effect in WT, there was no impact of C13 on LPS-stimulated IL-10 production (Figure 6O).

## 5 Discussion

### 5.1 AMPK negatively regulates the expression of proteins upregulated in pro-inflammatory macrophages

#### 5.1.1 Integration of AMPK-based and metabolite-based immunometabolic control

Compared with wild-type cells, AMPKα1 knockout macrophages caused increased expression of a wide range of proteins known to be upregulated in M1 macrophages.

IRG1 is the TCA metabolic enzyme that generates itaconate [56] and we found it was upregulated in AMPKα1 KO macrophages. Importantly, these data provide robust genetic evidence of cross-talk between metabolite-based (itaconate) and protein-kinase based (AMPK) immunometabolic control. Itaconate promotes stabilisation of NRF2 through alkylation of KEAP1, allowing NRF2 to increase expression of target genes with anti-oxidant and anti-inflammatory properties such as HO-1 [57,58]. Notably, HO-1 expression was suppressed in AMPKα1 KO macrophages. In addition, expression of the multifunctional adaptor protein p62, which regulates NRF2 via a non-canonical pathway [59] was altered in AMPKα1 KO macrophages, suggesting that AMPK regulates NRF2 activity by more than one mechanism.

Based on previous evidence in hepatocytes [60], AMPK deletion is likely to affect other aspects of metabolic regulation of the macrophage. PFKFB3 expression was strongly increased by LPS, and was increased in AMPKα1 KO compared to WT littermates. PFKFB3 is a critical glycolytic switch due to synthesis of fructose 2,6 bisphosphate, an allosteric activator of phosphofructokinase [61]. Despite these findings, metabolic changes were not identified between genotypes, except when unstimulated cells were exposed to oligomycin or FCCP, respectively to measure maximal glycolytic capacity and maximal respiratory capacity. AMPKα1 KO cells showed reductions in both glycolytic and respiratory capacity and also exhibited more proton leak. AMPK has previously been identified as an inducer of spare respiratory capacity [62] and our findings are consistent with this. Loss of spare respiratory capacity in the knockouts might suggest direct effects of knockout on respiration, as well as loss of AMPK dependent regulation of proton leak and/or the changes in glycolytic capacity. The functional significance of these changes will however require further investigation. Despite these differences, LPS had a much larger effect on respiratory chain protein abundance than did genotype. A further metabolic enzyme upregulated in LPS-stimulated AMPKα1 KO cells, CH25H, converts cholesterol to the antiviral metabolite 25-hydroxycholesterol (25-HC) [63,64]. 25-HC is thought to promote atherosclerosis, and was shown to induce foam cell formation and amplify inflammatory signalling through the transcription factor AP-1 [65].

Arginine metabolism plays a pivotal role in macrophage polarisation. Consistent with our observation of elevated levels of proteins associated with M1 macrophages in AMPKα1 KO cells, we also observed increased expression of the inducible arginine transporter CAT2, iNOS and ASS1, as well as elevated levels of NO production in AMPKα1 KO macrophages following LPS treatment. These data suggest that AMPK acts as a brake on arginine metabolism in M1 macrophages, limiting arginine uptake and nitric oxide production. It is plausible that this difference could contribute to the observed changes in respiratory capacity described above but WT and AMPKα1 KO macrophages were similarly able to switch from OXPHOS to glycolysis following LPS stimulation.

AMPKα1 KO also vastly upregulated key enzymes that control prostaglandin synthesis, including the rate-limiting enzymes COX1 and COX2. The increase in TBXAS and decrease in HPGDS under basal conditions will likely shift PGH_2_ metabolism from PGD_2,_ which is a vasodilator, to TXA_2_, which is a vasoconstrictor. MRP4, which regulates prostaglandin efflux, was also upregulated in the AMPKα1 KO macrophages. In functional analysis, we observed a large increase in PGE_2_ levels following LPS induction of KO macrophages confirmed with wild-type. Combined, these data suggest that AMPK acts to limit prostaglandin synthesis, efflux and production in M1 macrophages.

We also demonstrated that LPS induces the expression of the histidine transporter SLC15A3, which was higher in AMPKα1 KO than WT. SLC15A3 expression has been shown to influence TLR4-mediated IL-6 and TNF-α production [66]. In addition, the expression of HAL was robustly elevated in AMPKα1 KO compared to WT. HAL catalyses the non-oxidative deamination of histidine, the first step in histidine degradation.

Finally, NRAMP1, also upregulated in AMPKα1 KO cells in response to LPS, is an iron transporter induced in M1 polarisation probably by transcriptional control of HIF1a [67]. NRAMP1 maintains the supply of Fe^2+^ and Mn^2+^ ions during immune responses, and has been shown to play a critical role in host resistance to intracellular pathogens such as *Leishmania*, *Mycobacteria* and *Salmonella* [68].

#### 5.1.2 Established M1 markers are enhanced in AMPKα1 KO macrophages

In addition to modulation of immunometabolism, our data demonstrate that AMPK restrains the expression of a repertoire of proteins associated with pro-inflammatory macrophages. Several recognised M1 markers were increased in AMPKα1 KO cells, including costimulatory molecules CD86 [69] and CD40 [19]. Each of these molecules have been shown previously to be present both *in vivo* and in *in vitro* classically activated macrophages [70]. Consistent with AMPK expression and activation mediating distinct thresholds of immune activation, we have previously identified little effect of AMPK activation on LPS-induced CD40 expression [19]. CD86 is a ligand for CD28 and because its expression increases rapidly, is thought to be the major CD28 ligand during early T-cell activation [71]. CD40 is in the TNF receptor superfamily member and involved in atherogenesis triggered by CD4^+^ T-cells and in CD11c^+^ dendritic cells [72]. CD14 is a pattern recognition receptor that binds directly to LPS and acts as a co-receptor for TLR4, playing a critical role in LPS-stimulated responses [73,74].

ICAM-1 expression is known to be increased in M1 macrophages, where it is believed to act as a receptor for efferocytosis [33]. ICAM-1 may also suppress M2 polarisation of tumour-associated macrophages [75]. CD69 is a costimulatory molecule for T cell proliferation and activation that is expressed on several immune cell types including T cells, B cells, neutrophils and macrophages [76]. CD38 is an ectoenzyme thought to be involved in calcium signalling and NAD + metabolism, whose expression is strongly induced in M1 macrophages [26,77]. MARCO is a scavenger receptor that mediates phagocytosis [78]. Together, these findings highlight the potential for AMPK to limit the wider inflammatory response by playing a negative regulatory role in the expression of transmembrane proteins that participate in the immune response.

#### 5.1.3 Cytokines, chemokines and other secreted proteins enhanced by AMPKα1 KO

The production of multiple pro-inflammatory cytokines, chemokines and secreted proteins were enhanced in AMPKα1-deficient M1 macrophages compared to WT. IL-1α and IL-1β were both robustly upregulated in LPS-stimulated AMPKα1 KO macrophages. This is in agreement with previous studies showing that LPS-stimulated IL-1β expression is elevated in both AMPKα1-and AMPKβ1-deficient BMDMs [36]. Further work will be required to tease out any role of nitric oxide in this effect. Our data also point to AMPK as a regulator of chemokine synthesis in inflammatory macrophages. RANTES is a chemokine sensed by several immune cell types including T-cells and monocytes [79], and CXCL10 drives recruitment of T-cells, eosinophils, monocytes and NK cells [80]. Proteins that play a role in the progression of atherosclerosis such as PAI-1 and MMP-14 were also elevated in AMPKα1 KO macrophages. Foam cells high in MMP-14 have previously been shown to be play an important role in atherogenesis and it has been found at high levels in atherosclerotic plaques [81,82]. PAI-1 has been implicated in atherosclerosis formation in humans and pharmacological inhibition of this protein can supress atherogenesis [83]. In cell line studies it is induced by LPS, with knockdown experiments suggesting that it augments the signalling response and secretion of other effector molecules [84]. C3 is a critical component of the complement cascade; however recent findings have also implicated it in renal fibrosis [85]. In addition, SAA3 was strongly increased in LPS-stimulated AMPKα1 KO BMDMs compared to WT. SAA3 has been shown to play a pro-atherogenic role in animal models of atherosclerosis [86]. Collectively, our data suggest that AMPK restrains excessive production of pro-inflammatory cytokines, chemokines and other secreted proteins in classically activated macrophages.

### 5.2 Pharmacological AMPK activation provides an additional threshold of control of cytokine secretion

Having studied effect of AMPKα1 KO, we then went on to study the impact of AMPK activation on key cytokines produced by LPS-stimulated macrophages. Previously we showed that metformin reduced IL-6 and IL-12 secretion in M1 macrophages [19], whilst others have shown that IL-10 is increased [36]. We observed all three of these hallmarks of metformin action at the lower dose of metformin used in our current study, with similar results using the highly selective α1-selective AMPK activator C13 on IL-6 and IL-12, suggesting that these effects of metformin are AMPK-dependent, with the effect of metformin on IL-10 AMPK-independent. Taken together, these results provide genetic confirmation that AMPK expression and AMPK activation provide different levels of cytokine regulation tailored to each cytokine. Thus, in response to LPS IL-6 and IL-12p40 are decreased by AMPK expression and then suppressed further by AMPK activation. There are two levels of suppression. In contrast, IL-10 production is increased by AMPK expression but unaffected by AMPK activation, suggesting that this cytokine is regulated by AMPK in an AMPK-*activation independent* manner, findings that are consistent with previous data [10,36]. Findings with C13 were consistent with these scenarios for each cytokine.

## 6 Conclusion

In conclusion, our systematic investigation establishes for the first time: (i) integration of metabolite-based and enzyme-based immunometabolic control of inflammatory macrophage activation, particularly involving arginine metabolism, prostaglandin synthesis and (ii) discrete thresholds of control unlocked by pharmacological AMPK activation. In response to an inflammatory stimulus, AMPK acts as a brake on M1 macrophage function, with pharmacological AMPK activation providing a further layer of control at the level of cytokine secretion. Our results indicate that evolution of AMPK has enabled discrete thresholds of immunometabolic control to arise beyond that possible with metabolite-based control alone.

## Data Availability

Data will be made available on request.
