# Supplementary material for: AMPK integrates metabolite and kinase-based immunometabolic control in macrophages
Source: Mol Metab. 2022 Dec 28;68:101661. doi: 10.1016/j.molmet.2022.101661 (PMC9842865; doi:10.1016/j.molmet.2022.101661)
Supplement: Multimedia component 1 [file mmc1.zip › molmet_101661_Mol Metabolism SUPPLEMENTARY figs R3 _V2_mmc1.pptm]

## Slide 1
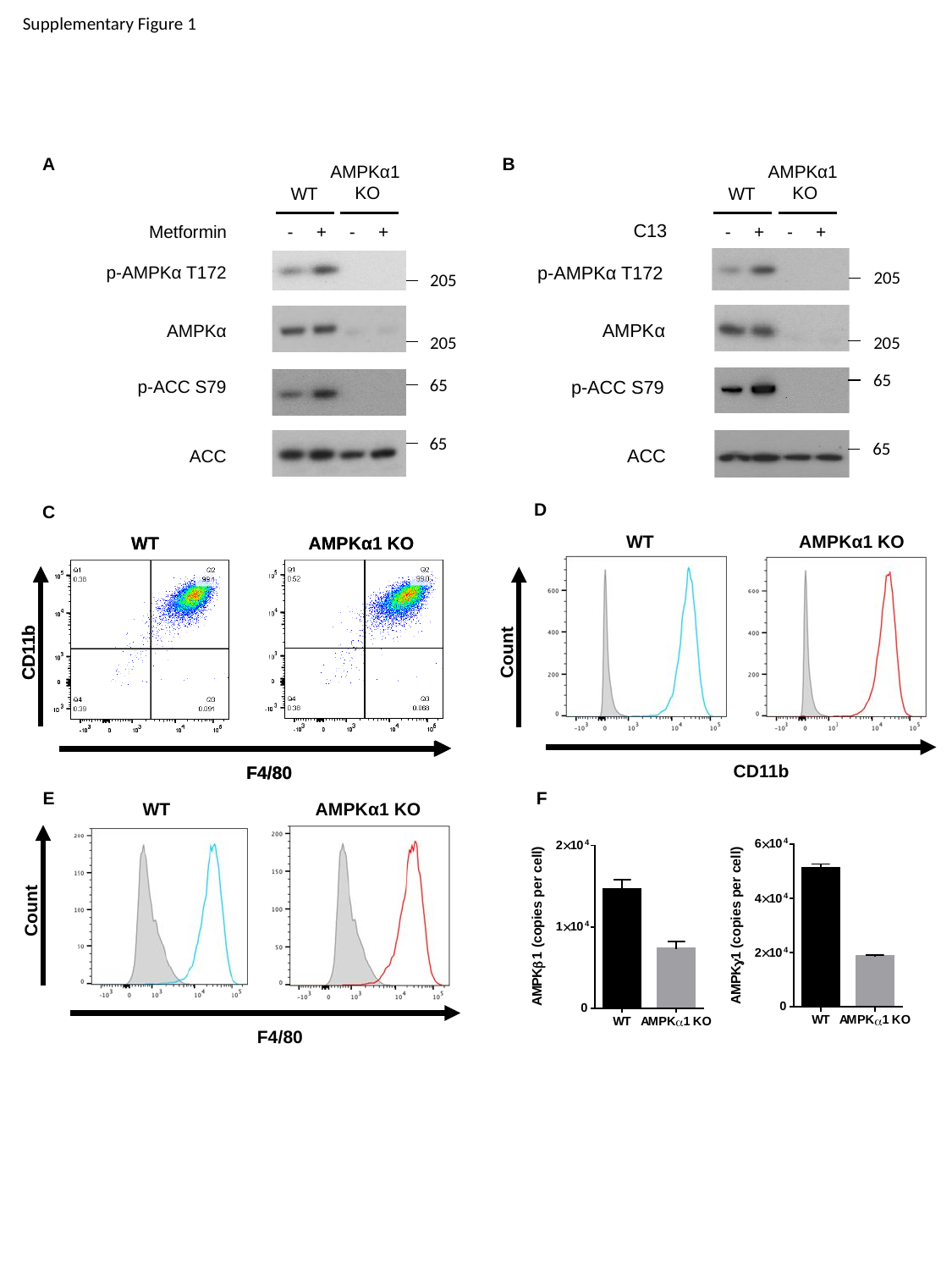

Supplementary Figure 1
B
A
AMPKα1
KO
AMPKα1
KO
WT
WT
C13
-
+
-
+
-
+
-
+
Metformin
p-AMPKα T172
p-AMPKα T172
205
205
AMPKα
AMPKα
205
205
65
65
p-ACC S79
p-ACC S79
65
65
ACC
ACC
D
C
WT
AMPKα1 KO
WT
AMPKα1 KO
CD11b
F4/80
WT
AMPKα1 KO
CD11b
F4/80
Count
CD11b
E
F
WT
AMPKα1 KO
Count
F4/80

## Slide 2
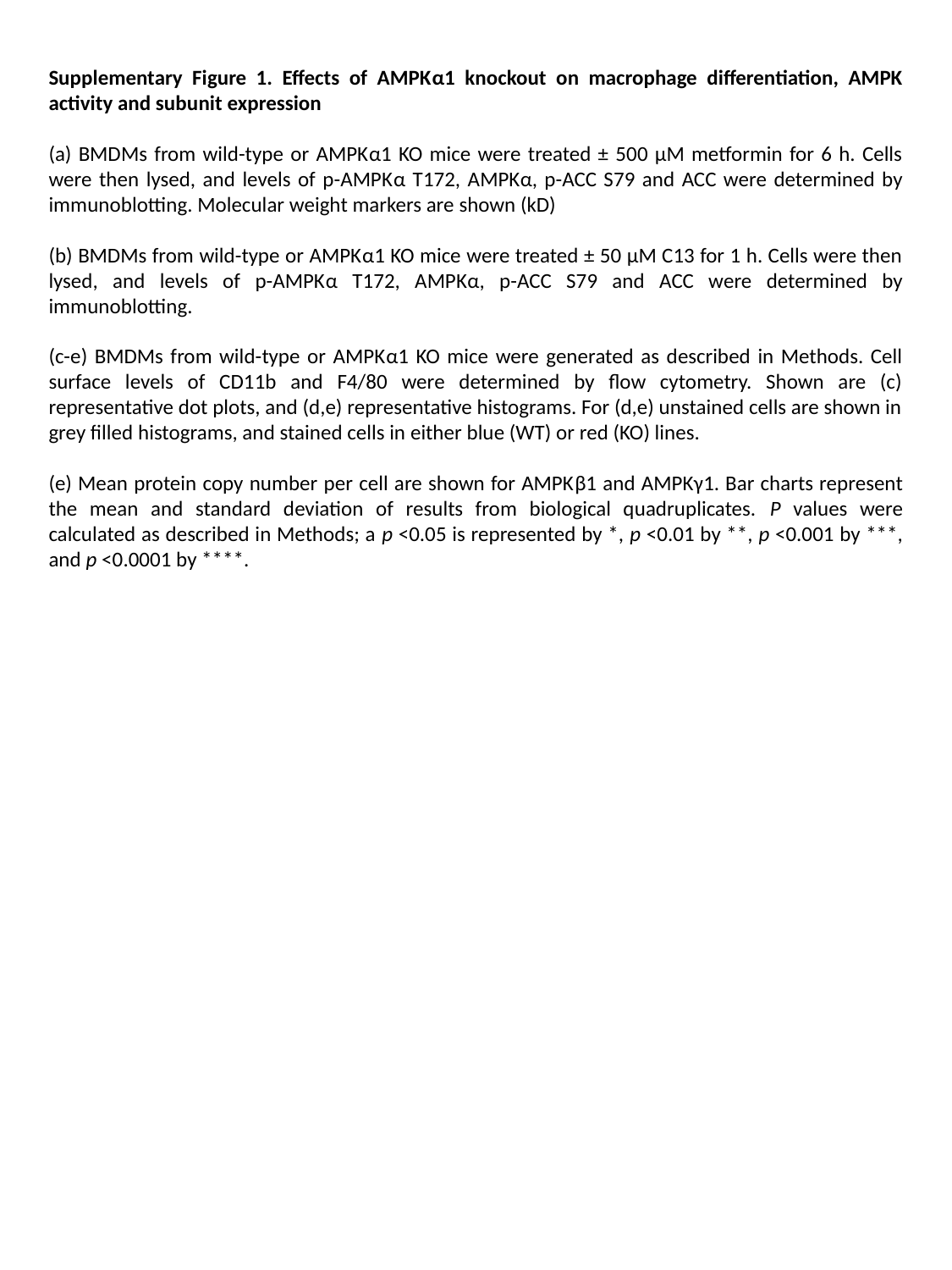

Supplementary Figure 1. Effects of AMPKα1 knockout on macrophage differentiation, AMPK activity and subunit expression
(a) BMDMs from wild-type or AMPKα1 KO mice were treated ± 500 µM metformin for 6 h. Cells were then lysed, and levels of p-AMPKα T172, AMPKα, p-ACC S79 and ACC were determined by immunoblotting. Molecular weight markers are shown (kD)
(b) BMDMs from wild-type or AMPKα1 KO mice were treated ± 50 µM C13 for 1 h. Cells were then lysed, and levels of p-AMPKα T172, AMPKα, p-ACC S79 and ACC were determined by immunoblotting.
(c-e) BMDMs from wild-type or AMPKα1 KO mice were generated as described in Methods. Cell surface levels of CD11b and F4/80 were determined by flow cytometry. Shown are (c) representative dot plots, and (d,e) representative histograms. For (d,e) unstained cells are shown in grey filled histograms, and stained cells in either blue (WT) or red (KO) lines.
(e) Mean protein copy number per cell are shown for AMPKβ1 and AMPKγ1. Bar charts represent the mean and standard deviation of results from biological quadruplicates. P values were calculated as described in Methods; a p <0.05 is represented by *, p <0.01 by **, p <0.001 by ***, and p <0.0001 by ****.

## Slide 3
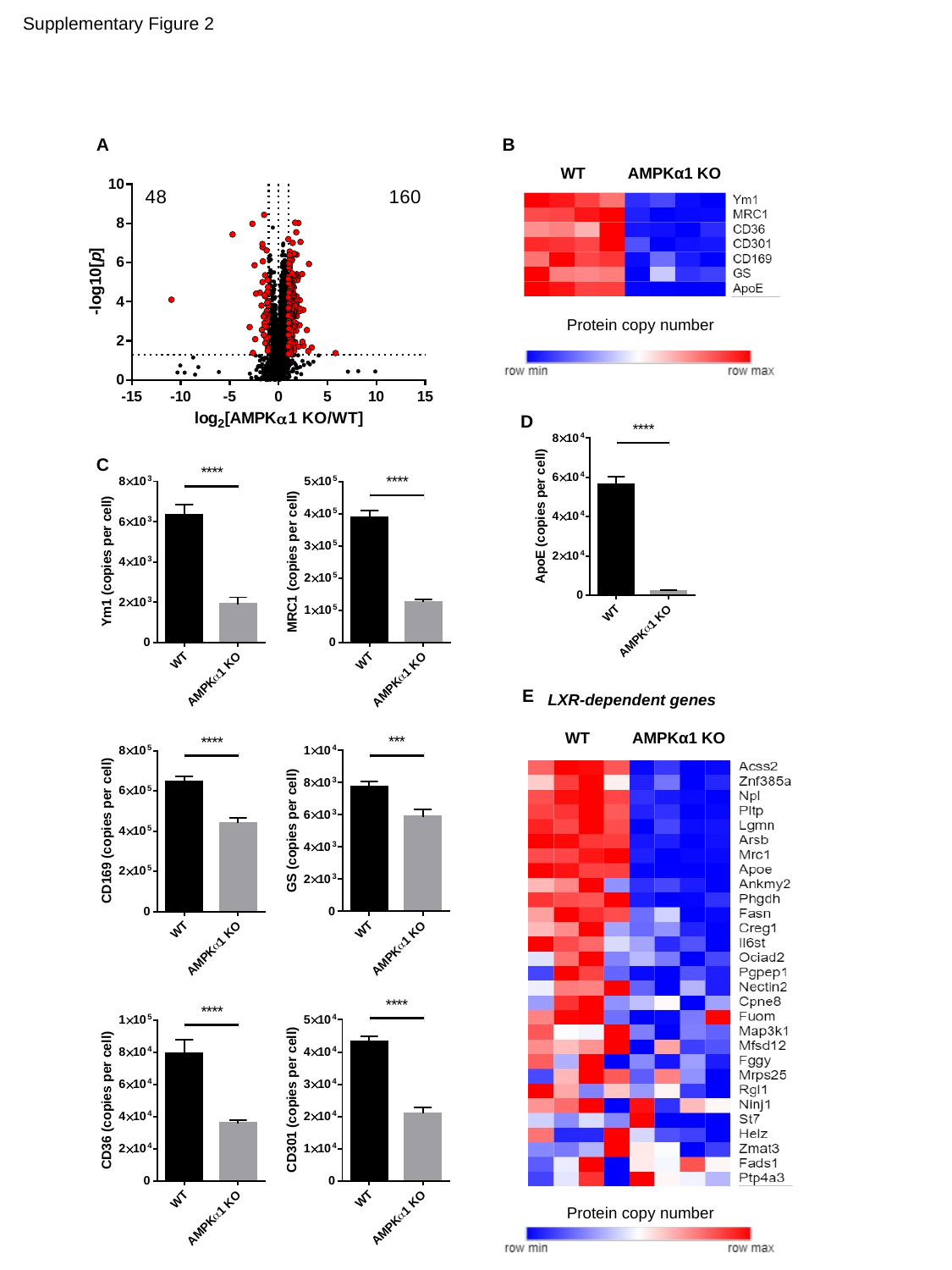

Supplementary Figure 2
A
B
WT
AMPKα1 KO
Protein copy number
D
C
E
LXR-dependent genes
WT
AMPKα1 KO
Protein copy number

## Slide 4
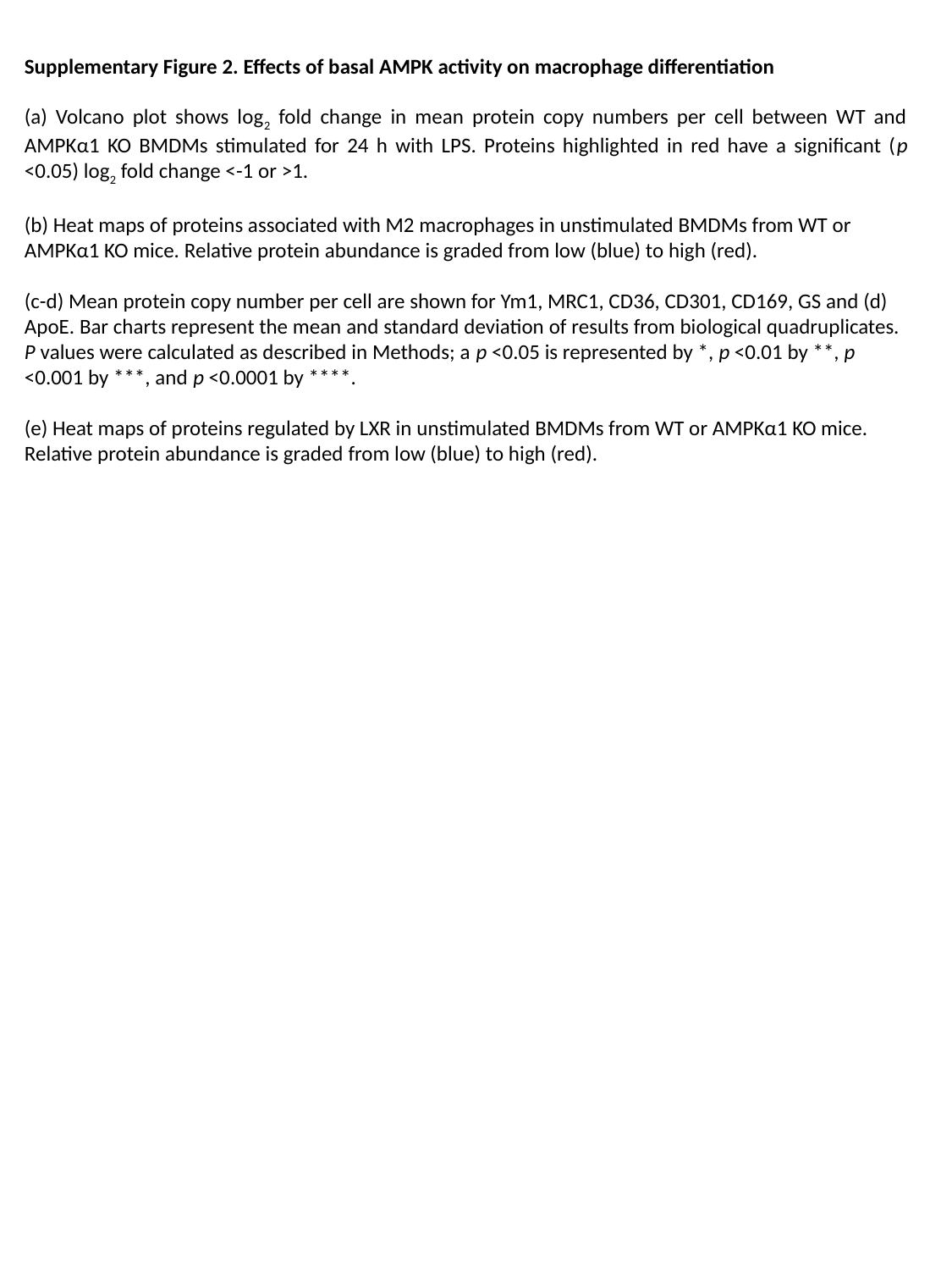

Supplementary Figure 2. Effects of basal AMPK activity on macrophage differentiation
(a) Volcano plot shows log2 fold change in mean protein copy numbers per cell between WT and AMPKα1 KO BMDMs stimulated for 24 h with LPS. Proteins highlighted in red have a significant (p <0.05) log2 fold change <-1 or >1.
(b) Heat maps of proteins associated with M2 macrophages in unstimulated BMDMs from WT or AMPKα1 KO mice. Relative protein abundance is graded from low (blue) to high (red).
(c-d) Mean protein copy number per cell are shown for Ym1, MRC1, CD36, CD301, CD169, GS and (d) ApoE. Bar charts represent the mean and standard deviation of results from biological quadruplicates. P values were calculated as described in Methods; a p <0.05 is represented by *, p <0.01 by **, p <0.001 by ***, and p <0.0001 by ****.
(e) Heat maps of proteins regulated by LXR in unstimulated BMDMs from WT or AMPKα1 KO mice. Relative protein abundance is graded from low (blue) to high (red).

## Slide 5
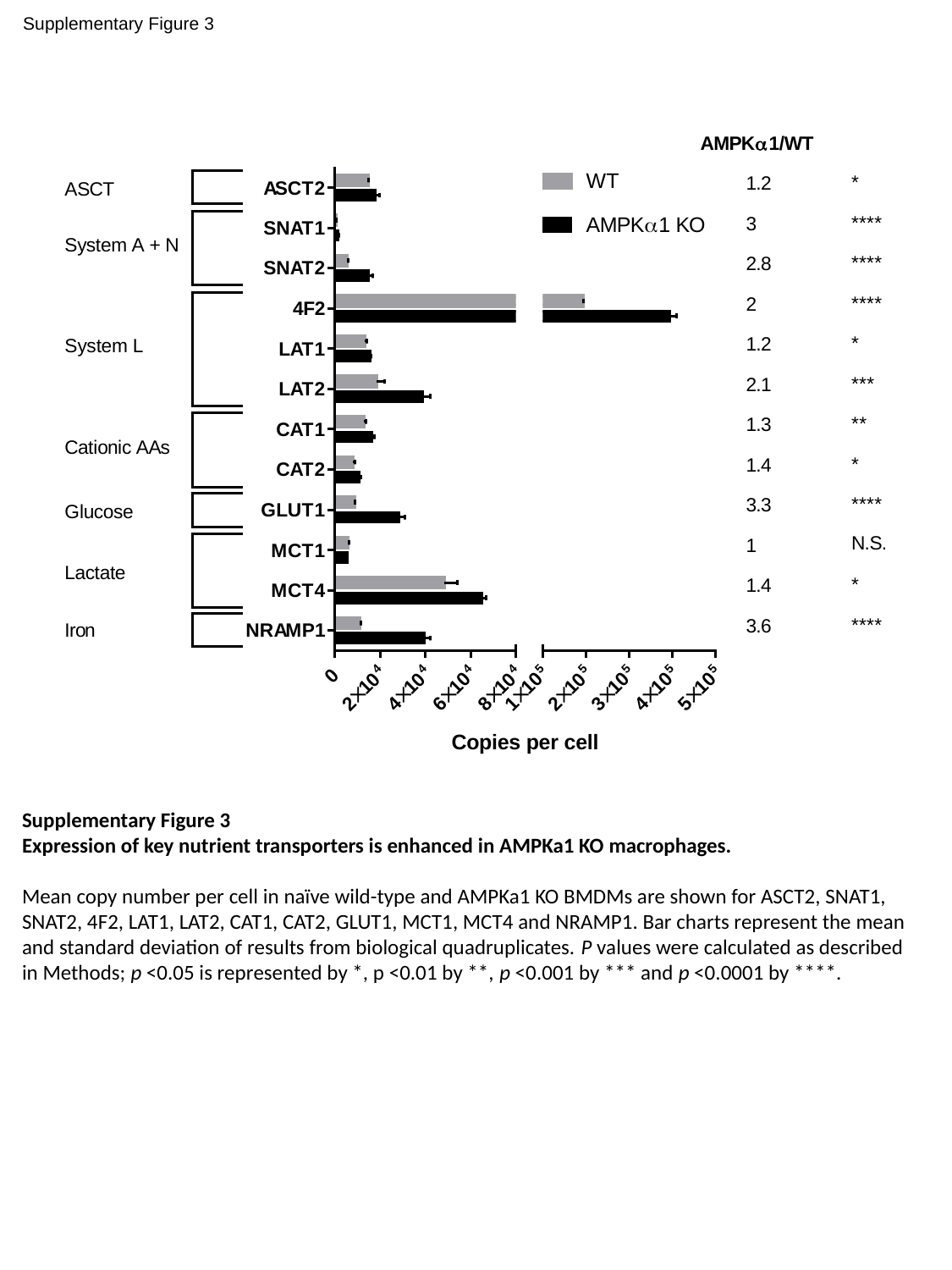

Supplementary Figure 3
Supplementary Figure 3
Expression of key nutrient transporters is enhanced in AMPKa1 KO macrophages.
Mean copy number per cell in naïve wild-type and AMPKa1 KO BMDMs are shown for ASCT2, SNAT1, SNAT2, 4F2, LAT1, LAT2, CAT1, CAT2, GLUT1, MCT1, MCT4 and NRAMP1. Bar charts represent the mean and standard deviation of results from biological quadruplicates. P values were calculated as described in Methods; p <0.05 is represented by *, p <0.01 by **, p <0.001 by *** and p <0.0001 by ****.

## Slide 6
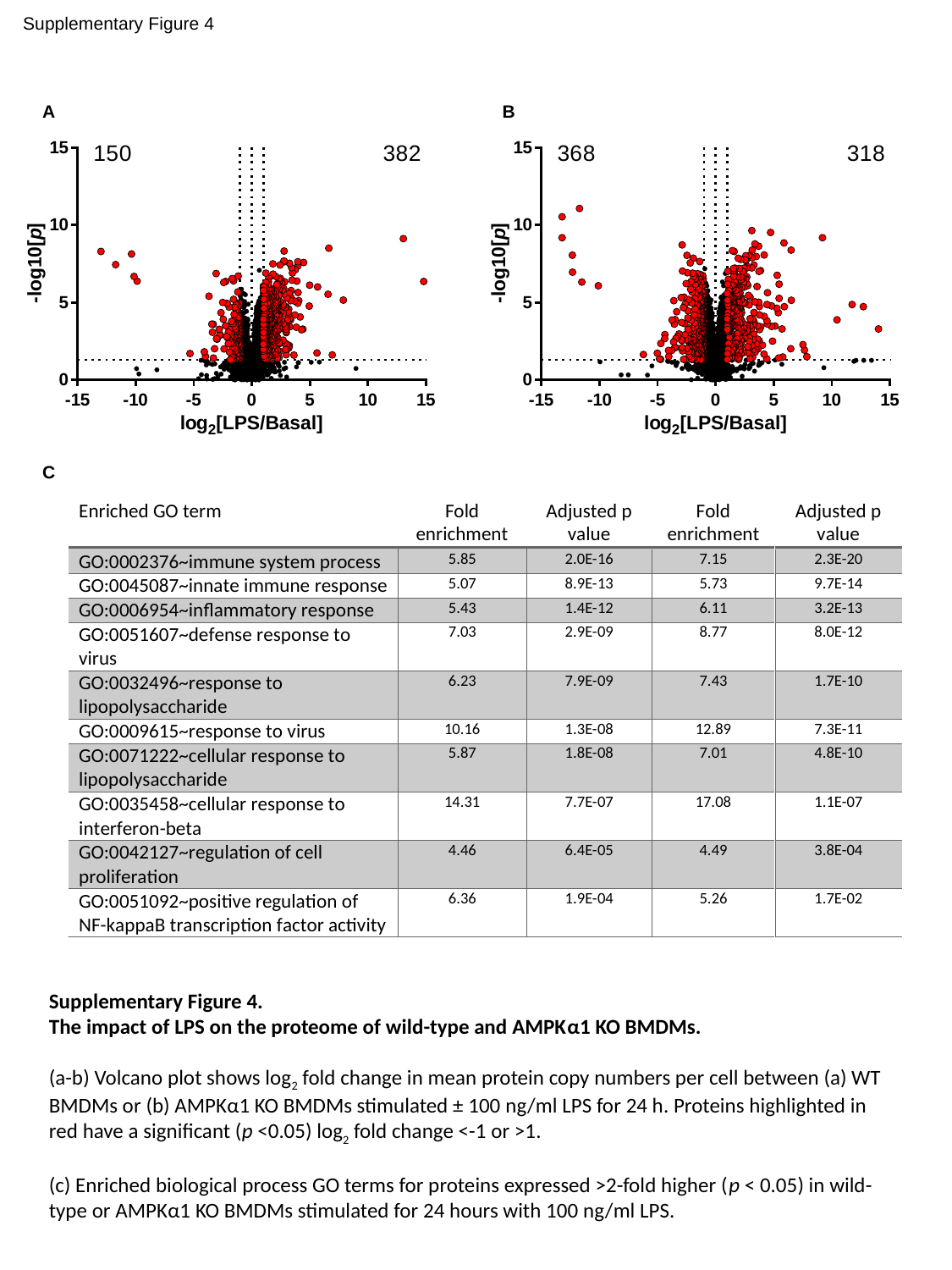

Supplementary Figure 4
B
A
C
Supplementary Figure 4.
The impact of LPS on the proteome of wild-type and AMPKα1 KO BMDMs.
(a-b) Volcano plot shows log2 fold change in mean protein copy numbers per cell between (a) WT BMDMs or (b) AMPKα1 KO BMDMs stimulated ± 100 ng/ml LPS for 24 h. Proteins highlighted in red have a significant (p <0.05) log2 fold change <-1 or >1.
(c) Enriched biological process GO terms for proteins expressed >2-fold higher (p < 0.05) in wild-type or AMPKα1 KO BMDMs stimulated for 24 hours with 100 ng/ml LPS.

## Slide 7
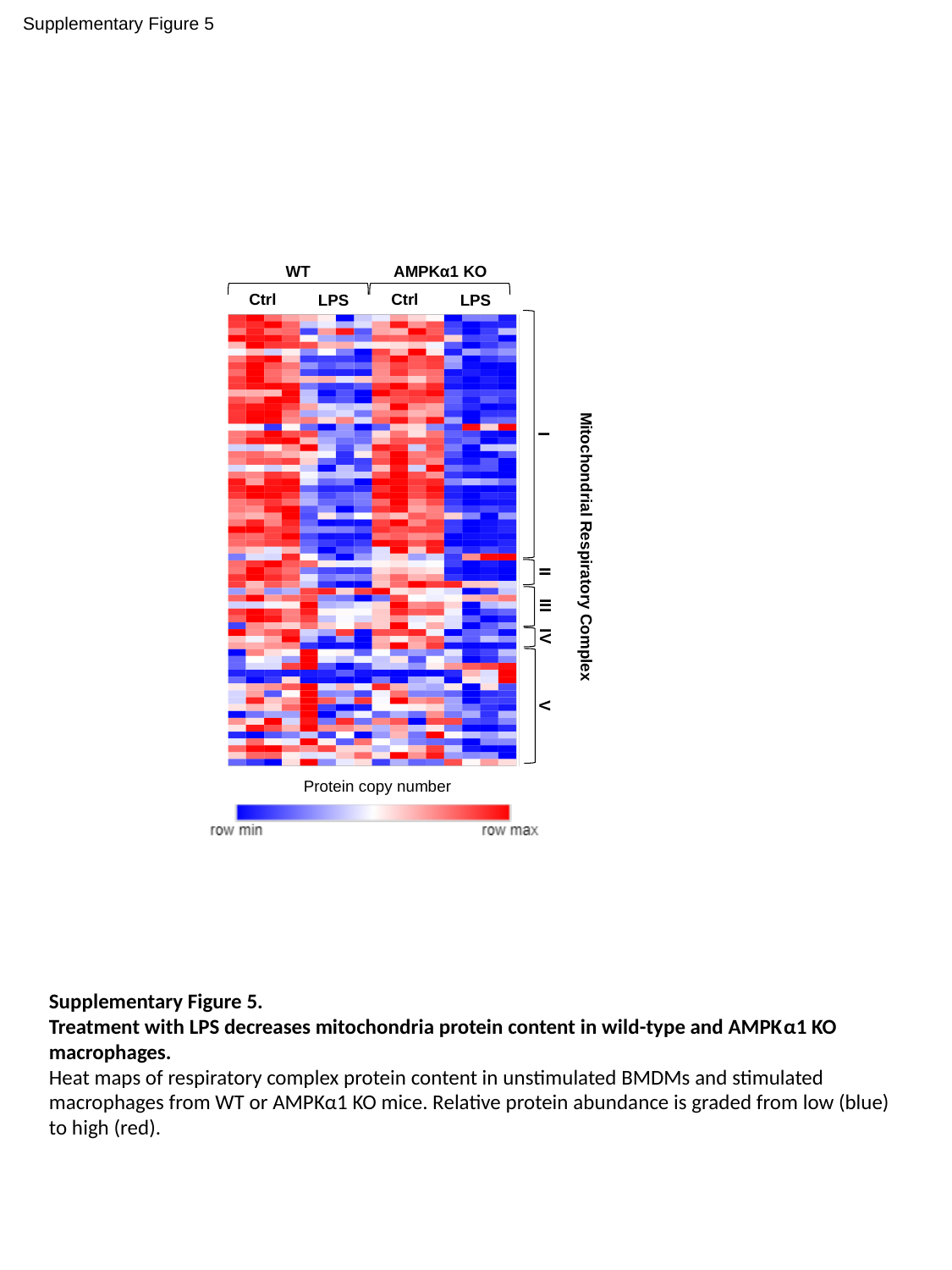

Supplementary Figure 5
WT
AMPKα1 KO
Ctrl
Ctrl
LPS
LPS
I
Mitochondrial Respiratory Complex
II
III
IV
V
Protein copy number
Supplementary Figure 5.
Treatment with LPS decreases mitochondria protein content in wild-type and AMPKα1 KO macrophages.
Heat maps of respiratory complex protein content in unstimulated BMDMs and stimulated macrophages from WT or AMPKα1 KO mice. Relative protein abundance is graded from low (blue) to high (red).
